# The Role of Apixaban in the Treatment of Heparin-Induced Thrombocytopenia

**DOI:** 10.7759/cureus.1428

**Published:** 2017-07-05

**Authors:** Sidra Khalid, Hamed Daw

**Affiliations:** 1 Internal Medicine, Fairview Hospital, Cleveland Clinic, USA; 2 Department of Hematology and Oncology, Fairview Hospital, Cleveland Clinic, USA

**Keywords:** apixaban, heparin induced thrombocytopenia, argatroban

## Abstract

Heparin-induced thrombocytopenia (HIT) can present as arterial and venous thrombosis in adults who are treated with heparin. We present a case of a patient who developed HIT when she was treated for deep venous thrombosis (DVT) and pulmonary embolism with heparin. During the treatment with heparin and while being transitioned to warfarin, she developed arterial thrombosis. A work-up for HIT was sent, and it was positive. She was started on the argatroban drip and her platelet counts stabilized. Since her platelet counts remained stable and were not increasing for three weeks, we decided to transition the patient to an oral anticoagulant. She was started on apixaban, a novel oral anticoagulant (NOAC), and her platelet counts remained stable. Therefore, through this case, we highlight the importance of platelet counts remaining stable when a patient with HIT is treated with apixaban.

## Introduction

Heparin-induced thrombocytopenia (HIT) occurs in 0.2% to 0.5% of adults who are treated with heparin [1]. HIT results from a decline in platelet count 5 to 10 days after starting heparin therapy. It can be treated by using argatroban infusion; after that, patients can be transitioned to warfarin [2]. Usually, the transition to warfarin is easy, but we present a patient with pulmonary embolism and deep venous thrombosis (DVT) who developed arterial thrombosis while on heparin and warfarin. This complication and a low platelet count caused us to use apixaban instead of warfarin. Currently, new oral anticoagulants (NOACs) are approved by the US Food and Drug Administration (FDA) for venous thromboembolism, and limited data are available for the management of HIT [2]. Therefore, we present a case that highlights the efficacy of using apixaban after a short course of argatroban infusion for HIT. 

## Case presentation

**Figure 1:**
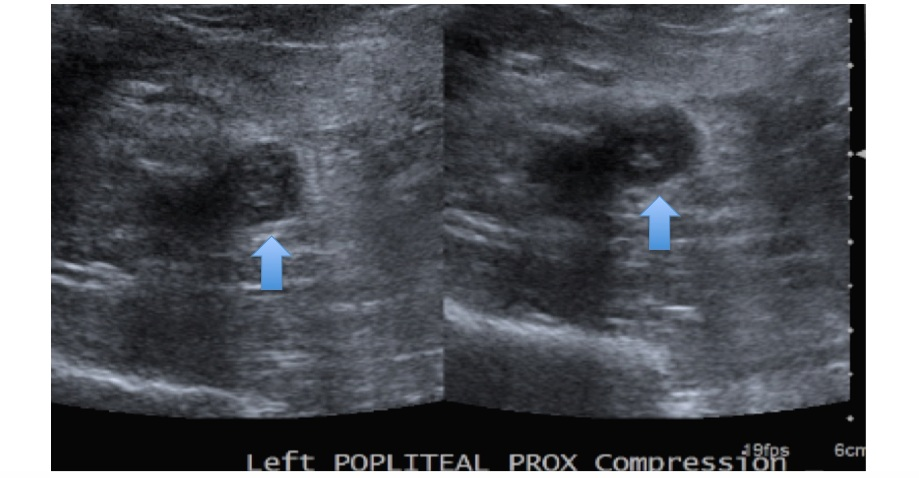
Ultrasound of DVT shows a noncompressible left popliteal vein

**Figure 2:**
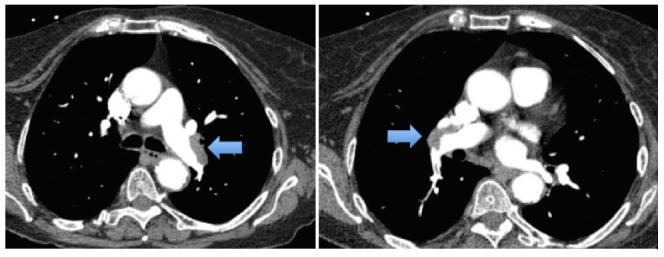
CT-chest: thrombi in the distal main right and left pulmonary arteries (arrows)

**Figure 3:**
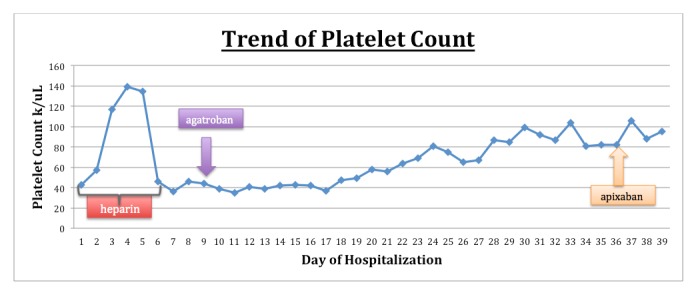
Trend of platelet count in the management of HIT

An 82-year-old female presented to the emergency department with pain in the chest and left leg. She had a nonradiating, left-sided, pressure-like chest pain of moderate intensity, which started after the left leg pain. Her past medical history was significant for hypertension, chronic kidney disease (CKD) stage 3, and chronic thrombocytopenia. Her platelet count had been decreasing from 160,000 uL for one year. She was hemodynamically stable and her physical examination was unremarkable. Her laboratory tests were significant for a platelet count of 43,000 uL, creatinine of 1.56 mg/dL, and a glomerular filtration rate (GFR) of 32 mL/min/1.73 m^2^. An ultrasound for DVT of the lower extremities showed acute DVT extending from the distal left superficial femoral vein to the left calf (Figure 1). A computed tomography (CT) scan of the chest with contrast showed thrombi in the distal main right and left pulmonary arteries in the right-upper, middle, and lower lobes and the left upper and lower lobes, signifying a moderate clot burden (Figure 2). Consequently, the patient received a heparin bolus (5100 U) and was started on a heparin drip for pulmonary emboli. On Day 3 of the heparin drip, her platelet count was 117,000 uL and she was started on warfarin due to the decreased GFR. On Day 6, the heparin drip was discontinued, as the international normalized ratio (INR) was therapeutic. On Day 7, the INR was 6.7, platelets had dropped to 47,000 uL, creatinine was 2.01 mg/dL, and she developed a painful, bluish discoloration of her left second toe, signifying ischemia. Warfarin was held and vitamin K was given. Her 4T score was calculated to be 7. The anti-platelet factor 4 was positive, 3.559 with optical density (OD) less than 0.400, and the heparin-induced platelet aggregation assay was positive, suggestive of HIT. She was started on the argatroban drip; fondaparinux was not started due to renal dysfunction. On the argatroban drip, her platelet count ranged from a low of 39,000 uL to a high of 104,000 uL and then became stable around 82,000 uL after three weeks. Since the platelet count remained at 88,000 uL on the argatroban drip, it was decided to transition her to apixaban (Figure 3). Therefore, argatroban was stopped and apixaban was started. Her platelet count slowly increased to 95,000 uL in three days and she was discharged home on apixaban. 

## Discussion

In the United States, approximately 10,000 cases of HIT occur annually. HIT is characterized by a decline in platelet count after initiating heparin. HIT is caused when there is platelet activation by anti-heparin platelet factor 4 antibodies. It can result in both arterial and venous thromboembolism. In clinical practice, the 4T score is used to assess the pretest probability of HIT [3]. HIT antibody testing is performed through immunoassays or functional assays to confirm a diagnosis of HIT [4].

Management includes immediate withdrawal of heparin and prompt initiation of non-heparin anticoagulants [1]. The drugs approved by the FDA for HIT are argatroban and bivalirudin. Argatroban is a parenteral direct thrombin inhibitor and is widely used for HIT. Bivalirudin is used mostly during cardiovascular interventions for patients with confirmed or suspected HIT.

Fondaparinux is a parenteral factor Xa inhibitor, which is recommended as an option for the management of HIT by the American College of Chest Physicians guidelines. Studies have shown fondaparinux’s rate of thrombotic and bleeding risks to be similar to that of argatroban. It should be used with caution in patients with renal dysfunction because it has a long half-life with no antidote [3]. The newer therapeutic agents for HIT are NOAC, dabigatran, apixaban, and rivaroxaban. From these, apixaban is a direct factor Xa inhibitor that is structurally unrelated to heparin; as a result, the HIT antibodies do not target the drug, and it does not cause platelet aggregation and activation with serotonin release [5]. Properties such as rapid onset of action, oral administration, ease of use, and lack of monitoring make this NOAC ideal for the management of HIT. In a retrospective study, Sharifi M et al. looked at 22 patients with HIT, who were treated with argatroban and then transitioned to NOAC (dabigatran-6, rivaroxaban-11, apixaban to five patients). These results demonstrated that a short course of argatroban followed by NOACs is highly safe and effective for treating HIT because it prevents thrombosis and stabilizes the platelet count [6]. Likewise, in our case, a trial of apixaban for three days improved the platelet count without any bleeding.

## Conclusions

Our case highlights the importance of using apixaban in patients with HIT in the setting of a thrombotic event and to stabilize platelet count. Further clinical trials are necessary to demonstrate the safety and efficacy of NOACs in treating HIT.
